# Influence of the Single Coronary Vessel on Acute Outcomes of In-Stent CTO Recanalization

**DOI:** 10.31083/j.rcm2307249

**Published:** 2022-06-29

**Authors:** Jan-Erik Guelker, Christian Blockhaus, Edward Kemala, Klaus Ingerfurth, Julian Kuervers, Alexander Bufe

**Affiliations:** ^1^Department of Cardiology and Rhythmology, Petrus Hospital, 42283 Wuppertal, Germany; ^2^Faculty of Health, University Witten/Herdecke, 58455 Witten, Germany; ^3^Department of Cardiology, Helios Clinic, 47805 Krefeld, Germany; ^4^Department of Cardiology, Johanna Etienne Hospital, 41462 Neuss, Germany

**Keywords:** in-stent chronic total occlusion, percutaneous coronary intervention, single coronary artery, acute outcome

## Abstract

**Objective:**

Recanalization of in-stent chronic total occlusion (IS-CTO) is challenging and has resulted in inconsistent results. The aim of our study was to analyze the influence of the individual coronary vessels on the acute outcomes following IS-CTO PCI.

**Methods:**

This was an observational retrospective study, including 66 patients undergoing recanalization of a CTO. The CTO interventions were performed bi-femoral using 7-French guiding catheters. A composite endpoint summarizing severe complications was evaluated, including emergency coronary artery bypass grafting surgery (CABG) and cardiac death.

**Results:**

We subdivided our cohort into three groups (LAD group, LCX group, RCA group). The retrograde technique and the utilization of an extension catheter were used more frequently in patients with a RCA IS-CTO. There was no significant difference between the composite safety endpoints amongst the three groups. Technical success was independent of the involved vessel.

**Conclusions:**

Success and complication rates are independent of the occluded vessel. This challenging and complex coronary intervention is feasible and can be carried out in complete safety.

## 1. Introduction

In interventional cardiology recanalization of chronic total occlusion (CTO) remains a complex procedure. A CTO of a coronary artery can be identified in up to 18% of patients undergoing coronary angiography [1]. Due to novel recanalization strategies and emerging devices, percutaneous coronary intervention (PCI) has become a promising treatment option [2, 3, 4, 5, 6, 7]. CTO revascularization has been shown to be a predictor for the prevention of cardiac death [8].

Particularly PCI of in-stent occlusions (IS-CTO) is extremely demanding, and studies have shown inconsistent results regarding outcomes and complications [9, 10, 11, 12, 13, 14, 15]. There is little data in the literature on outcomes amongst the three individual major coronary artery branches. IS-CTO is defined as a >50% stenosis of a previously stented segment.

In this study we want to analyze the influence of the individual coronary vessels on the acute outcomes following IS-CTO PCI.

## 2. Methods 

Our study is an observational study. 66 patients who underwent IS-CTO PCI between 2012 and 2021 were retrospectively included. The indication for CTO-PCI was based on the presence of clinically symptoms including typical angina pectoris or dyspnea [16, 17, 18].

The complex interventions were performed bi-femoral using 7-French guiding catheters. After initial contralateral contrast injections, the length of the CTO lesion and the existence and extent of collateral vessels was carefully analyzed. Antegrade and retrograde recanalization techniques were used. Heparin was administered during CTO-PCI guided by measurements of the activated clotting time (>300 sec).

After a successful recanalization a dual antiplatelet therapy consisting of aspirin and clopidogrel for at least 6 months was instituted. The primary endpoint was technical success, defined as a successful recanalization of the CTO with a residual stenosis less than 30% and restoration of thrombolysis in myocardial infarction (TIMI)-flow grade 3.

A composite endpoint was evaluated including acute cardiac death, vascular and bleeding complications, coronary perforation, which necessitated treatment by pericardial puncture, peri-interventional myocardial infarction (MI) which was defined as an increase in cardiac troponin blood levels in a patient who also exhibits signs or symptoms of MI, stroke and emergency coronary artery bypass grafting surgery (CABG) [19, 20].

## 3. Statistical Analysis

The distribution of continuous variables was characterized by mean ± standard deviation, or median with interquartile ranges (25th to 75th percentiles), as well as ranges (minimum to maximum), and the distribution of categorical variables by absolute and relative frequencies. The Shapiro-Wilk test was used to test for normality of the data. The Kruskal-Wallis test was used without testing normality because of the small sample size. Fisher’s exact test was used to test the differences in the distributions of categorical variables. According to the exploratory character of the analysis all *p* values were interpreted as descriptive measures rather than as definitive inferential measures. A *p* value of less than 0.05 was considered statistically significant, *p *< 0.10 was considered as a statistical trend.

## 4. Results

66 patients were included and subdivided into three groups (LAD group, LCX group, RCA group). 47 (72.2%) patients suffered from a RCA IS-CTO, 11 (16.7%) from a LAD IS-CTO and 8 (12.1%) from a LCX IS-CTO.

Table 1 shows the baseline clinical characteristics. The majority of the patients were male (87.9%) and 34.8% were older than 65 years of age. Patients in the LAD group had a higher incidence of diabetes mellitus (LAD group: 45.5%, LCX group: 25.0%, RCA group: 17.0%, *p* value: 0.147). Other cardiovascular risk factors such as smoking habits, increased cholesterol values, and a family history of coronary artery disease (CAD), were distributed equally amongst the groups. Hypertension was present in 90.9% of the LAD group, 62.5% in the LCX group and 68.1% in the RCA group (*p* value: 0.292). Patients in the LCX group had a higher incidence of prior coronary artery bypass graft (CABG) surgery (LAD group: 18.2%, LCX group: 37.5%, RCA group: 10.6%, *p* value: 0.115). Fewer patients in the LAD group had a prior CTO PCI attempt (LAD group: 36.4%, LCX group: 75.0%, RCA group: 72.3%, *p* value: 0.079). Almost all patients in each group had an ejection fraction (EF) greater than 40% (LAD group: 100%, LCX group: 100%, RCA group: 97.9%, *p* value: 1).

**Table 1.** **Baseline characteristics**.

	LAD	LCX	RCA	*p*-value*
	(n = 11)	(n = 8)	(n = 47)	
Age >65 years	2 (18.2%)	3 (37.5%)	18 (38.3%)	0.503
Male sex	10 (90.9%)	7 (87.5%)	41 (87.2%)	1.000
Diabetes	5 (45.5%)	2 (25.0%)	8 (17.0%)	0.147
COPD	0 (0.0%)	1 (12.5%)	2 (4.3%)	0.386
Smoking	3 (27.3%)	5 (62.5%)	21 (44.7%)	0.304
PAD	1 (9.1%)	1 (12.5%)	8 (17.0%)	1.000
Hypertension	10 (90.9%)	5 (62.5%)	32 (68.1%)	0.292
Family history for CAD	1 (9.1%)	1 (12.5%)	13 (27.7%)	0.375
Prior MI	7 (63.6%)	2 (25.0%)	16 (34.0%)	0.142
Prior CABG	2 (18.2%)	3 (37.5%)	5 (10.6%)	0.115
Prior CTO PCI attempt	4 (36.4%)	6 (75.0%)	34 (72.3%)	0.079
Prior PCI	9 (81.8%)	6 (75.0%)	32 (68.1%)	0.759
EF ≥40%	11 (100.0%)	8 (100.0%)	46 (97.9%)	1.000
Cholesterol >200 mg/dL	0 (0.0%)	2 (28.6%)	9 (24.3%)	0.538
HDL Cholesterol >40 mg/dL	5 (71.4%)	8 (100.0%)	25 (67.6%)	0.306
LDL Cholesterol >100 mg/dL	3 (42.9%)	4 (57.1%)	16 (43.2%)	0.901

*Fisher’s exact test; CABG, coronary artery bypass graft; CAD, coronary artery disease; COPD, chronical obstructive pulmonary disease; CTO, chronic total occlusion; EF, Ejection fraction; HDL, high density cholesterol; LAD, left anterior descending; LCX, left circumflex; LDL, low density lipoprotein; MI, myocardial infarction; PAD, peripheral artery disease; PCI, percutaneous coronary; RCA, right coronary artery.

Table 2 summarizes the angiographic parameters and peri-procedural characteristics.

**Table 2.** **Procedural and angiographic characteristics**.

		LAD	LCX	RCA	*p*-value*
		(n = 11)	(n = 8)	(n = 47)	
Coronary artery disease					0.392
	1 vessel	2 (18.2%)	2 (25.0%)	9 (19.1%)	
	2 vessel	4 (36.4%)	3 (37.5%)	24 (51.1%)	
	3 vessel	5 (45.5%)	3 (37.5%)	14 (29.8%)	
Blunt Stump		5 (45.5%)	7 (87.5%)	30 (63.8%)	0.180
Calcification		9 (81.8%)	6 (75.0%)	33 (70.2%)	0.905
Bending >90°		5 (45.5%)	2 (25.0%)	30 (63.8%)	0.089
J-CTO Score >3		5 (45.5%)	4 (50.0%)	25 (53.2%)	0.926
Retrograde technique		2 (18.2%)	0 (0.0%)	16 (34.0%)	0.101
Use of an extension catheter		1 (9.1%)	0 (0.0%)	29 (61.7%)	0.041
Use of IVUS		9 (81.8%)	6 (75.0%)	35 (74.5%)	0.926
Length of lesion**		40 mm (10–60)	25 mm (15–70)	40 mm (15–80)	0.182
Fluoroscopy time**		43 min (12–48)	31 min (12–65)	42 min (11–97)	0.407
Examination time**		110 min (50–150)	85 min (45–115)	120 min (35–240)	0.141
Amount of contrast medium**		300 mL (100–500)	210 mL (100–450)	250 mL (120–630)	0.555
Number of stents**		3 (1–5)	2 (1–3)	2 (1–4)	0.364
Diameter of stents**		3.0 mm (2.5–3.5)	3.0 mm (2.75–3.5)	3.5 mm (2.5–4.0)	0.037
Length of stent**		66 mm (30–120)	46 mm (23–69)	66 mm (18–132)	0.212
Complication rate		0 (0.0%)	1 (12.5%)	1 (2.1%)	0.255
Technical success rate		11 (100.0%)	7 (87.5%)	40 (85.1%)	0.495

*Fisher’s exact test; **median (min-max); IVUS, Intravascular ultrasound; J-CTO, japanese chronic total occlusion; LAD, left anterior descending; LCX, left circumflex; RCA, right coronary artery.

**Fig. 1.**
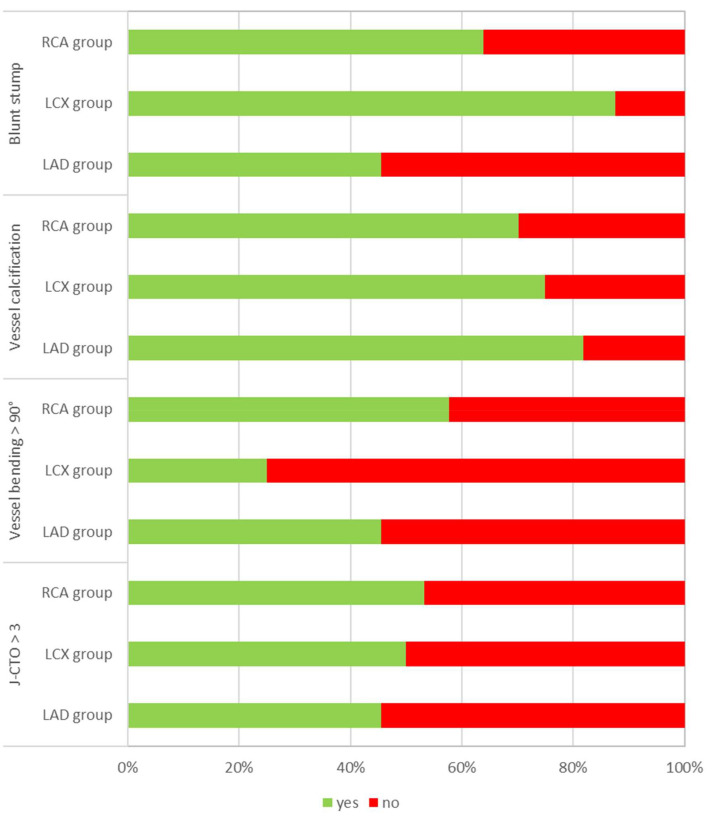
**Distribution of the J-CTO, blunt stump morphology, vessel calcification and vessel bending**.

The J-CTO score, including the degree of calcification of the occlusion, bending over 90° in the occluded segment, a blunt stump morphology, the length of the occluded segment longer than 20 mm and a previously failed recanalization attempt, was comparable in all groups; however, the bending of the vessel was more frequent in the RCA group (Fig. 1). The retrograde technique was used more frequently in patients with a RCA IS-CTO (LAD group: 18.2%, LCX group: 0.0%, RCA group: 34.0%, *p* value: 0.10). The use of a GuideLiner (Vascular Solutions Inc., Minneapolis, MN, USA) was used more frequently in the RCA group and the diameter of the implanted stents were larger in these patients (LAD group: 3.0 mm, LCX: 3.0 mm, RCA: 3.5 mm, *p* value: 0.037).

The number of stents (LAD group: 3, LCX group: 2, RCA group: 2, *p* value: 0.364) and the length of stents (LAD group: 66 mm, LCX group: 46 mm, RCA group: 66 mm, *p* value: 0.212) were similar amongst the groups.

Technical success was independent of the involved vessel (LAD group: 100%, LCX group: 87.5%, RCA: 85.1%).

Acute procedural complications derived from the composite endpoint were rare and showed no significant difference between the groups (LAD group: 0.0%, LCX group: 12.5%, RCA group: 2.1%, *p* value: 0.255). They included mostly vascular complications such as a local hematoma at the puncture site and could be treated without further consequences. No severe complications such as peri-procedural death, stroke or MI occurred (Table 3).

**Table 3.** **In-hospital clinical events**.

	LAD	LCX	RCA	*p*-value*
	(n = 11)	(n = 8)	(n = 47)	
In-hospital death	0 (0.0%)	0 (0.0%)	0 (0.0%)	1.000
Hamatoma	0 (0.0%)	1 (12.5%)	1 (2.1%)	0.255
Stroke	0 (0.0%)	0 (0.0%)	0 (0.0%)	1.000
Perforation	0 (0.0%)	0 (0.0%)	0 (0.0%)	1.000

*Fisher’s exact test; LAD, left anterior descending; LCX, left circumflex; RCA, right coronary artery.

## 5. Discussion

Several studies have reported the challenges involved in IS-CTO recanalization. A large registry could demonstrate the incidence of a IS-CTO PCI was 11% [21]. Mir *et al*. [10] emphasized that PCI for IS-CTO was associated with higher odds of MACE and MI compared to PCI for de-novo CTO. Data by Guan *et al*. [22] stressed that effectiveness and safety of this complex procedure are reasonable, but the risk of cardiac death and MI is higher among patients with IS-CTO. In another study including 81 patients, Gao *et al*. [23] added that patients with IS-CTO had worse Seattle Angina Questionnaire scores concerning anginal stabilities than the patients with de novo CTO.

Karmpaliotis *et al*. [24] provided some reasons for the difficulties to recanalize IS-CTO which may explain the on-going challenges involved with this complex procedure. It was particularly emphasized that the previous use of under-expanded stents is one key problem. This leads to the problem that the wire may enter the subintimal space. Furthermore, a possible stent fracture may complicate the wiring even with stiff wires. This is even more complex in tortuous vessels which are often associated with hard fibrous tissue and high calcium content [24]. In contrast to data from Azzalini *et al*. [13], we experienced many In-Stent CTO in the RCA.

This present study emphasizes three important findings:

First, the retrograde technique was used more frequently in patients with a RCA IS-CTO. We know that the retrograde approach, when used by experienced operators who have been well trained, can produce higher retrograde success in complex CTO lesions [25]. Surmely *et al*. [26] proposed that septal collaterals of the LAD can be used as an optimal access for the retrograde approach in RCA-CTO revascularization.

Second, the bending of the vessel was more frequent in the RCA group. That is consistent with the fact that the use of an extension catheter, such as the GuideLiner, was more frequently required. Several studies have proposed that a mother-and-child catheter is a simple, safe and efficacious adjunctive device for complex CTO recanalization when despite standard measures, it is not possible to deliver an initial balloon or microcatheter across the occluded segment [27, 28, 29, 30]. It helps to avoid implantation of under-expanded stents which contributes to in-stent occlusion. Furthermore, information about the amount of neointimal or peri-stent calcium, and multiple old stent strut layers are important determinants of new stent under expansion which is associated with adverse long-term outcomes [31].

Third, we demonstrated that the technical success and complication rate in IS-CTO PCI is independent of the target vessel. To the best of our knowledge, this is the first study to document that there is no influence of the target vessel on the success of this complex and challenging intervention.

## 6. Study Limitations

Our study has some limitations. First, the study is an observational retrospective analysis and all the data was collected from one single center. Second, our study only reported in-hospital outcomes without a follow-up. Third, no data was available on the type of stent used in this cohort. Fourth, there was no clinical event adjudication by a clinical events committee. Fifth, all procedures were performed at one experienced PCI center, thus limiting the generalizability of our findings to centers with limited experience. Sixth, our cohort included only 66 patients.

## 7. Conclusions

Recanalization of in-stent CTO lesions is a challenging procedure in interventional cardiology. Success and complication rates are independent of the occluded vessel. Recanalization is safe and feasible when performed safely in experienced hands.
